# Validity of the MoCA as a cognitive screening tool in epilepsy: Are there implications for global care and research?

**DOI:** 10.1002/epi4.12991

**Published:** 2024-06-14

**Authors:** Anny Reyes, Bruce P. Hermann, Divya Prabhakaran, Lisa Ferguson, Dace N. Almane, Jerry J. Shih, Vicente J. Iragui‐Madoz, Aaron Struck, Vineet Punia, Jana E. Jones, Robyn M. Busch, Carrie R. McDonald

**Affiliations:** ^1^ Department of Radiation Medicine & Applied Sciences University of California San Diego California USA; ^2^ Department of Neurology University of Wisconsin School of Medicine and Public Health Madison Wisconsin USA; ^3^ Epilepsy Center Neurological Institute, Cleveland Clinic Cleveland Ohio USA; ^4^ Department of Neuroscience University of California San Diego California USA; ^5^ Department of Neurology Cleveland Clinic Cleveland Ohio USA; ^6^ Department of Psychiatry University of California San Diego California USA

**Keywords:** aging, cognitive screeners, cognitive taxonomy, global neuropsychology

## Abstract

**Objective:**

This study evaluated the diagnostic performance of a widely available cognitive screener, the Montreal cognitive assessment (MoCA), to detect cognitive impairment in older patients (age ≥ 55) with epilepsy residing in the US, using the International Classification of Cognitive Disorders in Epilepsy (IC‐CoDE) as the gold standard.

**Methods:**

Fifty older adults with focal epilepsy completed the MoCA and neuropsychological measures of memory, language, executive function, and processing speed/attention. The IC‐CoDE taxonomy divided participants into IC‐CoDE *Impaired* and *Intact* groups. Sensitivity and specificity across several MoCA cutoffs were examined. Spearman correlations examined relationships between the MoCA total score and clinical and demographic variables and MoCA domain scores and individual neuropsychological tests.

**Results:**

IC‐CoDE impaired patients demonstrated significantly lower scores on the MoCA total, visuospatial/executive, naming, language, delayed recall, and orientation domain scores (Cohen's *d* range: 0.336–2.77). The recommended MoCA cutoff score < 26 had an overall accuracy of 72%, 88.2% sensitivity, and 63.6% specificity. A MoCA cutoff score < 24 yielded optimal sensitivity (70.6%) and specificity (78.8%), with overall accuracy of 76%. Higher MoCA total scores were associated with greater years of education (*p* = 0.016) and fewer antiseizure medications (*p* = 0.049). The MoCA memory domain was associated with several standardized measures of memory, MoCA language domain with category fluency, and MoCA abstraction domain with letter fluency.

**Significance:**

This study provides initial validation of the MoCA as a useful screening tool for older adults with epilepsy that can be used to identify patients who may benefit from comprehensive neuropsychological testing. Further, we demonstrate that a lower cutoff (i.e., <24) better captures cognitive impairment in older adults with epilepsy than the generally recommended cutoff and provides evidence for construct overlap between MoCA domains and standard neuropsychological tests. Critically, similar efforts in other regions of the world are needed.

**Plain Language Summary:**

The Montreal cognitive assessment (MoCA) can be a helpful tool to screen for cognitive impairment in older adults with epilepsy. We recommend that adults 55 or older with epilepsy who score less than 24 on the MoCA are referred to a neuropsychologist for a comprehensive evaluation to assess any changes in cognitive abilities and mood.

**Key Points**

- Cognitive impairment in epilepsy is a pressing global concern given its impact on social outcomes, mood, and quality of life.
- We examined the sensitivity and validity of the MoCA in a group of older adults with epilepsy.
- A cutoff <24 provided optimal sensitivity and specificity in detecting cognitive impairment based on a validated cognitive taxonomy.
- Given that neuropsychology is underdeveloped in many regions of the world, there is a need to validate screening assessments that are sensitive and widely accessible including residing in low‐to‐middle‐income countries.

## 1 INTRODUCTION

Epilepsy is a prevalent global neurological disorder impacting over 50 million people,^1^, ^2^ with older adults representing the fastest growing segment of individuals with epilepsy.^3^ As a global disease, it is overrepresented in middle and especially lower income countries (LMIC) where a significant treatment gap exists not only in regard to the treatment of epilepsy but also in regard to the recognition, diagnosis, and treatment of cognitive and behavioral/psychiatric conditions.^4^ While the presence and treatment of seizures in older adults can lead to age‐accelerated cognitive change, epilepsy can also be complicated by the presence of cognitive dysfunction antecedent to the first recognized seizure, diagnosis, and treatment.^3^ Further, older adults with epilepsy are at increased risk of developing dementia, including Alzheimer's disease (AD), with studies reporting an up to a 3‐fold increased risk.^5^ Given the risk of cognitive abnormality, cognitive decline, and dementia, the International League Against Epilepsy (ILAE) taskforce on epilepsy in the elderly recommended routine cognitive–behavioral screening for all older adults with epilepsy.^6^

Unfortunately, there is a paucity of research aimed at validating the ability of brief cognitive screening tools to identify cognitive impairment (i.e., preclinical and frank dementia) in older adults with epilepsy. Studies of this type necessarily examine the diagnostic efficiency performance (e.g., sensitivity, specificity, positive and negative predictive power) of a screening test compared to gold standard neuropsychological measures. This is even a more pressing need for LMIC where neuropsychology, as a still developing clinical science, is scarcely available^7^, ^8^ and efficient accurate mental status assessment techniques are needed. Therefore, there is a critical need to validate the applicability of cognitive screening tests to epilepsy in general, with an eye to identifying culturally sensitive methods for screening cognition that can also be applied to populations in regions of the world with limited resources.

Several cognitive screening measures exist (e.g., MMSE, MoCA, EpiTrack, Neuropsychiatry Unit Cognitive Assessment Tool, Dementia Rating Scale, MiniCog, Community Screening Instrument for Dementia), but in order to facilitate the development of a common global nomenclature, classification, and non‐fragmented literature in epilepsy—a metric applicable across languages and cultures is a key consideration. In this regard, the MoCA appears an attractive option as it has been translated widely (i.e., nearly 100 languages) with associated training and quality control procedures (https://mocacognition.com), and with growing use internationally across multiple health conditions,^9^ especially in LMIC (Table S1). Furthermore, the MoCA has been shown to be more sensitive to detecting cognitive impairment in epilepsy compared to other screeners^10^, ^11^ given its inclusion of multiple cognitive domains unlike other measures that may primarily test memory (e.g., MMSE). Additionally, the MoCA enjoys generally broad use and familiarity not only in the general population but in several neurological disorders as well,^12^, ^13^, ^14^ so a formal extension to epilepsy, particularly late‐onset epilepsy, is a reasonable consideration.

To be integrated into the cognitive assessment of older adults with epilepsy and the broader epilepsy‐neuropsychology literature, it becomes imperative to understand the relationship of the MoCA to traditional neuropsychological tests and practice considered to be gold standard. A first step would be to compare MoCA outcomes (e.g., intact vs. impaired cognition) to an existing cognitive taxonomic system such as the International Classification of Cognitive Disorders in Epilepsy (IC‐CoDE). The IC‐CoDE was developed by the ILAE Neuropsychology Task Force to classify cognitive phenotypes based on patterns of cognitive impairment across cognitive domains.^14^ Comparison of MoCA to IC‐CoDE cognitive classification as *Intact* versus *Impaired* would address issues of sensitivity and specificity at a broad level and determine the most appropriate impairment cutoff for older adults with epilepsy. A second and more detailed comparison would be to examine the relationship of specific MoCA items (e.g., delayed memory) to widely used traditional neuropsychological tests (e.g., Rey Auditory Verbal Learning Test delayed recall), again to determine the external validity of MoCA items.

To that end, a battery of commonly used neuropsychological tests as well as the MoCA were administered to a sample of older adults with epilepsy (≥55 years of age) to address the following questions: First, what is the overall accuracy of the MoCA compared to a contemporary cognitive taxonomic approach (i.e., IC‐CoDE)? Second, what is the optimal cutoff to detect cognitive dysfunction in older adults with epilepsy? Third, what are the associations between specific MoCA items (e.g., memory, naming) and traditional neuropsychological measures of those abilities? Finally, what is the impact of important sociodemographic and clinical factors on MoCA performance?

## 2 METHODS

### 2.1 Participants

This study was approved by Institutional Review Boards at the University of California, San Diego (UCSD), Cleveland Clinic (CC), and University of Wisconsin‐Madison (UWM). Written informed consent was obtained from all patients. Patients were recruited as part of the BRain, Aging, and Cognition in Epilepsy (BrACE) study—a prospective longitudinal study of cognitive and brain aging in older adults with epilepsy. Inclusion criteria were: ≥55 years of age, diagnosis of focal epilepsy by a board‐certified neurologist with expertise in epileptology in accordance with the criteria defined by the ILAE,^15^ no history of prior therapeutic epilepsy neurosurgery (e.g., resection, laser ablation, neuromodulation device), and proficiency in English to complete cognitive testing.

### 2.2 Clinical, sociodemographic, and neuropsychological measures

Sociodemographic variables were obtained from patient self‐report and clinical records. All patients, regardless of recruitment site, completed an identical comprehensive neuropsychological assessment as part of a prospective, longitudinal study designed to evaluate cognitive changes in older adults with epilepsy compared to those with mild cognitive impairment and AD. As a result, the test battery included the MoCA (English 7.1), the Alzheimer's Disease Assessment Scale–Cognitive Subscale (ADAS‐Cog), and standard neuropsychological measures of memory, language, executive function, and processing speed. Table S2 includes the full list of neuropsychological tests, demographic corrections, and normative data. MoCA total score was corrected for education by adding one point to the total score for patients with less than 12 years of education.^16^ All neuropsychological scores were converted into T‐scores for ease of interpretability.

### 2.3 Application of IC‐CoDE

The IC‐CoDE taxonomy consists of five cognitive domains including memory, language, attention/processing speed, executive function, and visuospatial abilities. However, given the lack of availability of visuospatial tests in our battery, we used the four‐domain classification taxonomy that includes all the above domains but visuospatial.^14^ The IC‐CoDE phenotype classification is based on the number of impaired domains (at least two impaired tests), with single‐domain defined as impairment in one cognitive domain, bi‐domain defined as impairment in two domains, generalized defined as impairment in three or more domains, and intact defined as no impairment in any cognitive domain. We used <1 standard deviation below the normative mean as the impairment cutoff given that it has been shown to balance sensitivity and stability of impairment when examining profiles of scores^17^ and has been previously used in studies with older adults with epilepsy.^18^, ^19^ Given that the purpose of this study was to examine the MoCA's ability to detect cognitive impairment based on the IC‐CoDE classification (impaired vs. intact), patients with either single, bi‐domain, or generalized impairments were combined into one group (i.e., IC‐CoDE Impaired), whereas as those with no impaired domains were classified as IC‐CoDE Intact.

### 2.4 Statistical analyses

Independent *t*‐tests, Fisher's exact, and Kruskal–Wallis tests were conducted to compare clinical, demographic, and MoCA data. Cohen's *d* effect sizes are reported for differences in MoCA scores between groups. We examined the recommended MoCA cutoff score of <26 and alternative cutoff scores examined in prior studies in other clinical conditions and the general population.^20^, ^21^ Test measures including specificity, sensitivity, positive predictive value (PPV), negative predictive value (NPV), Youden Index, and Kappa statistic were calculated to evaluate agreement between MoCA impairment classification and the IC‐CoDE. Sensitivity was defined as the percentage of patients who were classified as IC‐CoDE *impaired* and who scored below each of the MoCA cutoffs. Specificity was defined as the percentage of patients who were classified as IC‐CoDE *intact* and who scored at or above each of the MoCA cutoffs. Receiver operating characteristic (ROC) curves were plotted to calculate the area under the curve (AUC). Discriminant function analyses (DFA) were conducted to examine whether individual MoCA domain scores could correctly classify impairment based on the IC‐CoDE (i.e., IC‐CoDE *intact* vs. *impaired*). Spearman rho correlations were conducted to examine the association between MoCA total score and demographic and clinical variables and between MoCA domain scores and standardized neuropsychological tests. To further explore these associations, a principal component analysis (PCA) with a varimax rotation using the five MoCA domain scores (attention, abstraction, naming, language, and memory) and the neuropsychological tests were conducted as a post hoc analysis. Significant results are reported with both an alpha = 0.05 and with Benjamini–Hochberg false discovery rate correction. All data were normally distributed based on skewness (<3) and kurtosis (<10). All analyses were conducted on IBM SPSS Statistics version 29, and figures were created using R version 4.1.

## 3 RESULTS

### 3.1 Patient sample

Fifty patients were included in the final sample. The cohort was on average 66.60 (SD = 6.88) years old, had on average 15.08 (SD = 2.67) years of education, was mostly Non‐Hispanic white (88%), and was mostly female (52%). On average, age of seizure onset was 43 years (SD = 20.49), duration of epilepsy was 23.6 years (SD = 18.63), and they were taking 1.73 (SD = 0.836) antiseizure medications (ASM). Patients in our cohort had overall lower MoCA total scores (epilepsy average = 24.66, SD = 3.31), compared to a sample of 365 cognitively normal older adults from the Alzheimer's Disease Neuroimaging Initiative (ADNI) database (control average = 26.05, SD = 2.68); *t*(413) = 3.34, *p* < 0.001 (Cohen's *d* = 0.462).

### 3.2 Clinical, demographic, and MoCA profiles between IC‐CoDE groups

**FIGURE 1:**
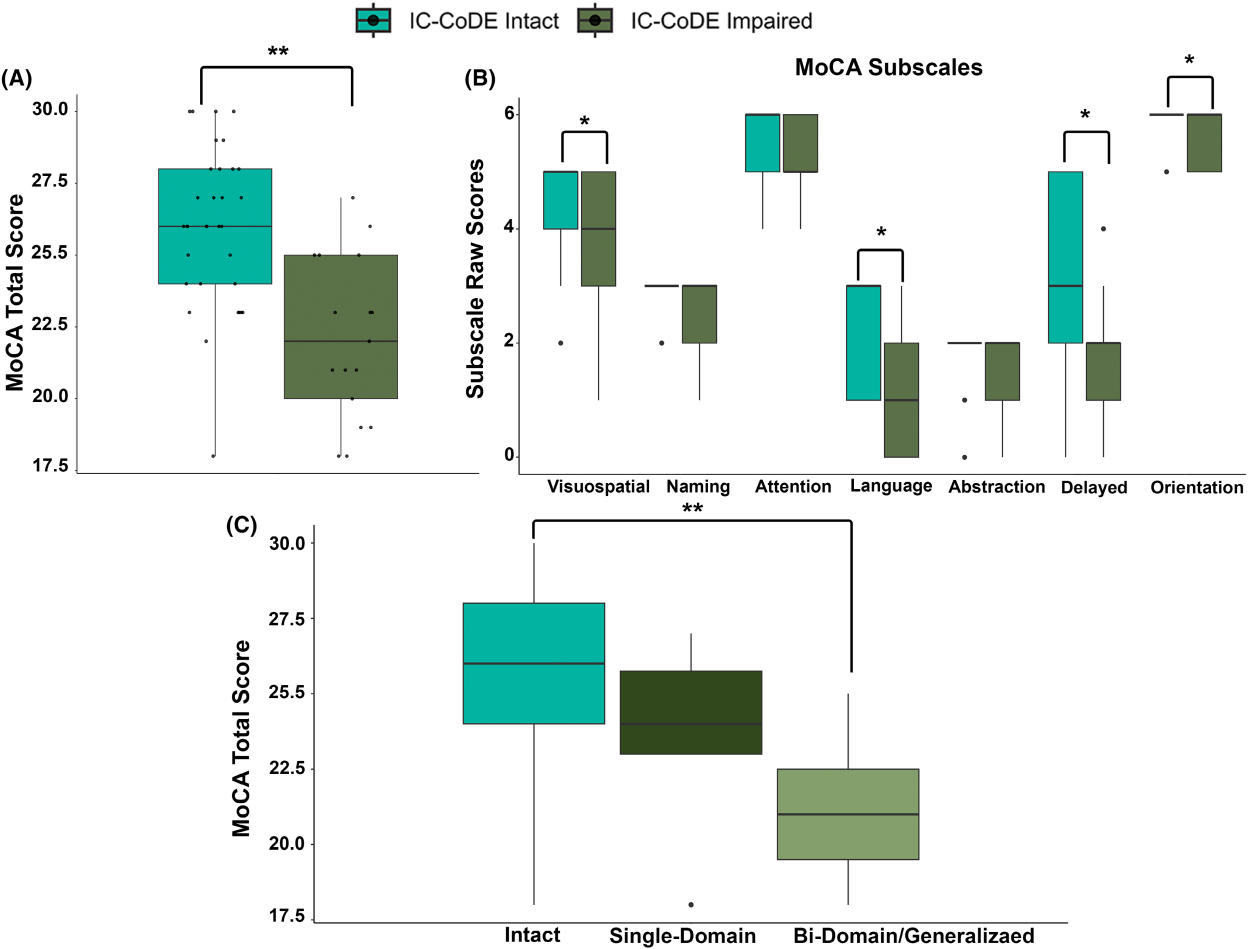
(A, B) Differences in MoCA total scores and domain scores between IC‐CoDE *intact* and *impaired* groups. (C) Distribution of MoCA total scores as a function of IC‐CoDE phenotype. Two asterisks represent significance <0.001 and one asterisk represents significance <0.05.

Based on standard neuropsychology measures and using a cutoff of <1 standard deviation below the normative mean, 34% of patients were classified as IC‐CoDE *impaired* (12% generalized, 10% bi‐domain, and 12% single domain). The most impaired domain was memory (range across tests: 14%–49%), followed by executive function (range across tests: 24%–26%), processing speed (range across tests: 14%–24%), and language (range across tests: 6%–24%). There were no differences in clinical or demographic variables between the IC‐CoDE groups (Table 1). Figure 1A,B shows the distribution of MoCA total and domain scores between IC‐CoDE *intact* and *impaired* groups. There were differences in MoCA total score (Cohen's *d* = 2.77), visuospatial/executive domain (Cohen's *d* = .935), naming domain (Cohen's *d* = 0.475), language domain (Cohen's *d* = 0.981), delayed recall domain (Cohen's *d* = 1.55), and orientation domain (Cohen's *d* = 0.336), with the IC‐CoDE *impaired* group having lower scores. There were no differences in attention (Cohen's *d* = 0.697) or abstraction (Cohen's *d* = 0.589). We also examined MoCA total score as a function of IC‐CoDE phenotype membership (Figure 1C). The bi‐domain/generalized showed lowered scores compared to the intact phenotype (*H* = 4.08, *p* < 0.001).

**TABLE 1:** Demographic, clinical, and MoCA characteristics between IC‐CoDE impaired and intact Groups.

	IC‐CoDE impaired	IC‐CoDE intact	Statistics	*p*‐Value
*N*	17 (34%)	33 (66%)		
Age: years	66.88 (7.57)	66.45 (6.62)	−0.206	0.838
Education: years	15.0 (2.55)	15.12 (2.77)	0.150	0.441
Sex: Female (%)	9 (47.1%)	15 (45.5%)	–	0.767
Race: White	14 (82.4%)	30 (93.8%)	–	0.255
Ethnicity: Hispanic (%)	0 (0%)	2 (6.1%)	–	0.542
Age at seizure onset	43.59 (20.03)	42.70 (21.03)	0.443	0.886
Epilepsy duration	23.29 (17.46)	23.76 (19.47)	0.467	0.935
Side				
Left	4 (23.5%)	17 (51.5%)	–	0.059
Right	1 (5.9%)	6 (18.2%)		
Bilateral	6 (35.3%)	5 (15.2%)		
Unknown	6 (35.3%)	5 (15.2%)		
Localization				
Temporal	9 (52.9%)	17 (51.5%)	–	0.962
Frontal	0 (0%)	2 (6.1%)		
Frontotemporal	4 (23.5%)	8 (24.2%)		
Unknown	6 (18.2%)	4 (23.5%)		
Number of ASM	2 (.816)	1.61 (.827)	−1.57	0.123
Type of ASM				
Levetiracetam	5	14	–	–
Lamotrigine	7	12		
Lacosamide	5	7		
Oxcarbazepine	4	5		
Zonisamide	2	2		
Topiramate	1	2		
Other^a^	7	9		
MoCA total score	22. 11 (2.8)	25.97 (2.74)	**4.66**	**<0.001**
Visuospatial/Executive	3.88 (1.11)	4.52 (.834)	**2.27**	**0.028**
Naming	2.59 (.712)	2.91 (.292)	**1.78**	**0.045**
Attention	5.24 (.831)	5.52 (.619)	1.35	0.092
Language	1.41 (1.17)	2.24 (.867)	**2.84**	**0.003**
Abstraction	1.59 (.712)	1.73 (.517)	0.790	0.217
Delayed recall	1.65 (1.32)	3.06 (1.66)	**3.05**	**0.002**
Orientation	5.71 (.470)	5.94 (.242)	**1.92**	**0.034**

*Note*: Bold: Significant at <0.05.

Abbreviations: ASM, antiseizure medication; IC‐CoDE, International Classification of Cognitive Disorders in Epilepsy; MoCA, Montreal Cognitive Assessment.

^a^ Other: clobazam, clonazepam, gabapentin, pentobarbital, phenobarbital, phenytoin, and midazolam.

### 3.3 Relationship between MoCA and IC‐CoDE classification

We examined impairment classification using the recommended MoCA cutoff of 26 (Table 2). There was moderate agreement with the IC‐CoDE classification with an overall 72% accuracy. Based on this cutoff, 54% of the sample was classified as impaired. Among those classified as impaired based on the MoCA, 55.5% were IC‐CoDE *impaired* and 44.5% were IC‐CoDE *intact*. Patients that were misclassified did not differ in age, education, sex, and race/ethnicity from those that were correctly classified (all *p*‐values >0.05). Based on the DFA, the individual MoCA domains correctly classified 78% of the cases for the IC‐CoDE taxonomy, misclassifying 23.5% of IC‐CoDE *impaired* patients and 21.2% of IC‐CoDE *intact* patients and having an overall 40% impairment classification rate.

**TABLE 2:** Classification accuracy across alternative MoCA cutoffs.

	MoCA defined impairment	Accuracy	Sensitivity	Specificity	PPV	NPV	Kappa	Youden index
MoCA <28	80%	54%	94.1%	42.4%	45.71%	93.33%	0.291	0.365
MoCA <27	70%	60%	100%	30.3%	42.5%	100%	0.228	0.303
MoCA <26	54%	72%	88.2%	63.6%	55.56%	91.3%	0.454	0.518
MoCA <25	44%	70%	70.6%	69.7%	54.54%	82.14%	0.376	0.403
**MoCA < 24**	**38%**	**76%**	**70.6%**	**78.8%**	**63.16%**	**83.87%**	**0.480**	**0.494**
MoCA <23	22%	80%	52.9%	93.9%	81.82%	79.49%	0.513	0.468
MoCA <22	18%	80%	47.1%	97.0%	88.89%	78.0	0.497	0.441

*Note*: Kappa: agreement between MoCA cutoff impairment classification and IC‐CoDE Impairment classification; Bold signifies optimal cutoff. Youden Index: (Sensitivity + Specificity) − 1.

Abbreviations: PPV, positive predictive value; NPV, negative predictive value.

### 3.4 Performance of alternative MoCA cutoffs in detecting IC‐CoDE‐defined impairment

**FIGURE 2:**
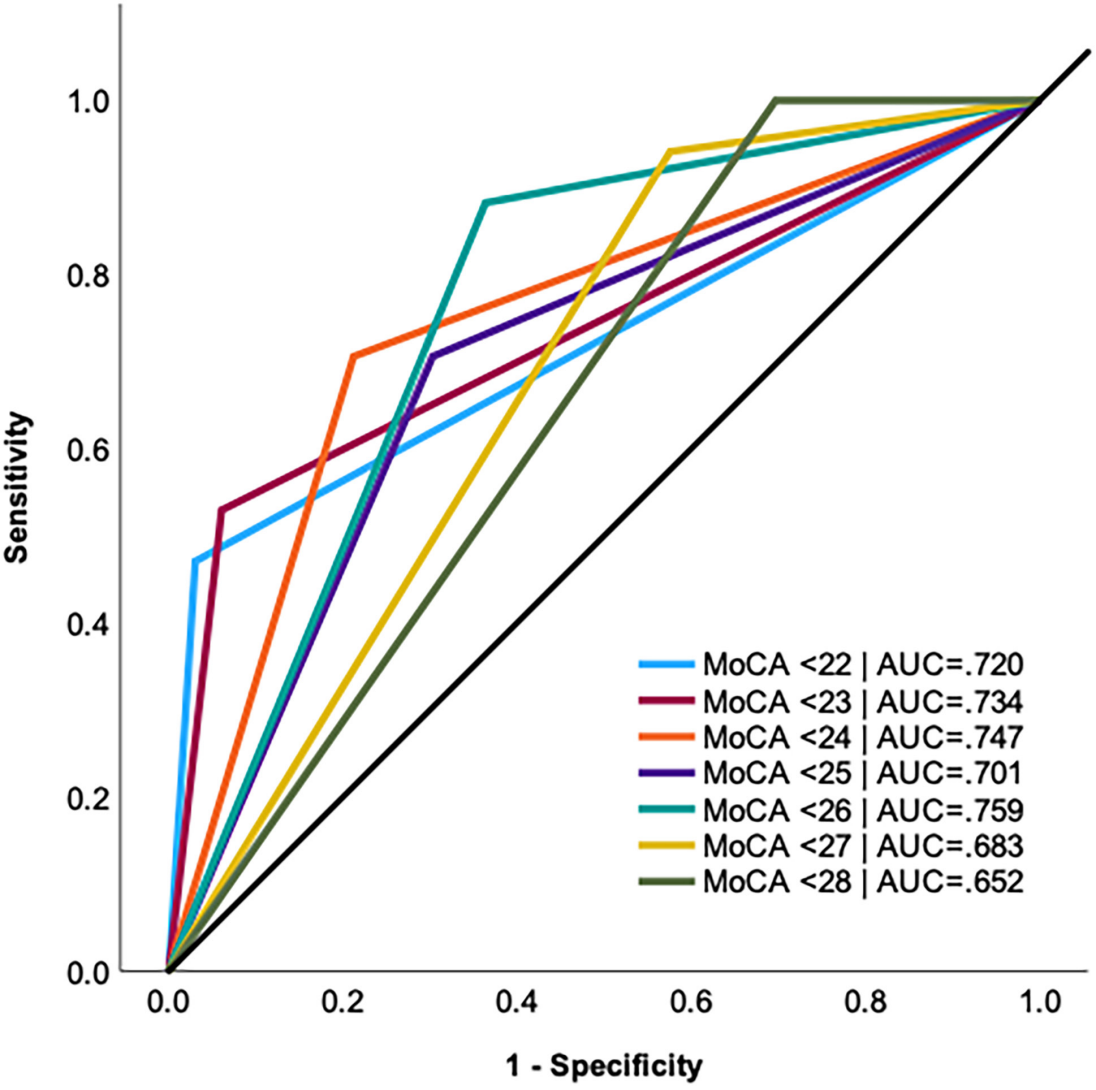
Receiver operating characteristic (ROC) curve across different MoCA cutoffs. AUC, area under the curve.

**FIGURE 3:**
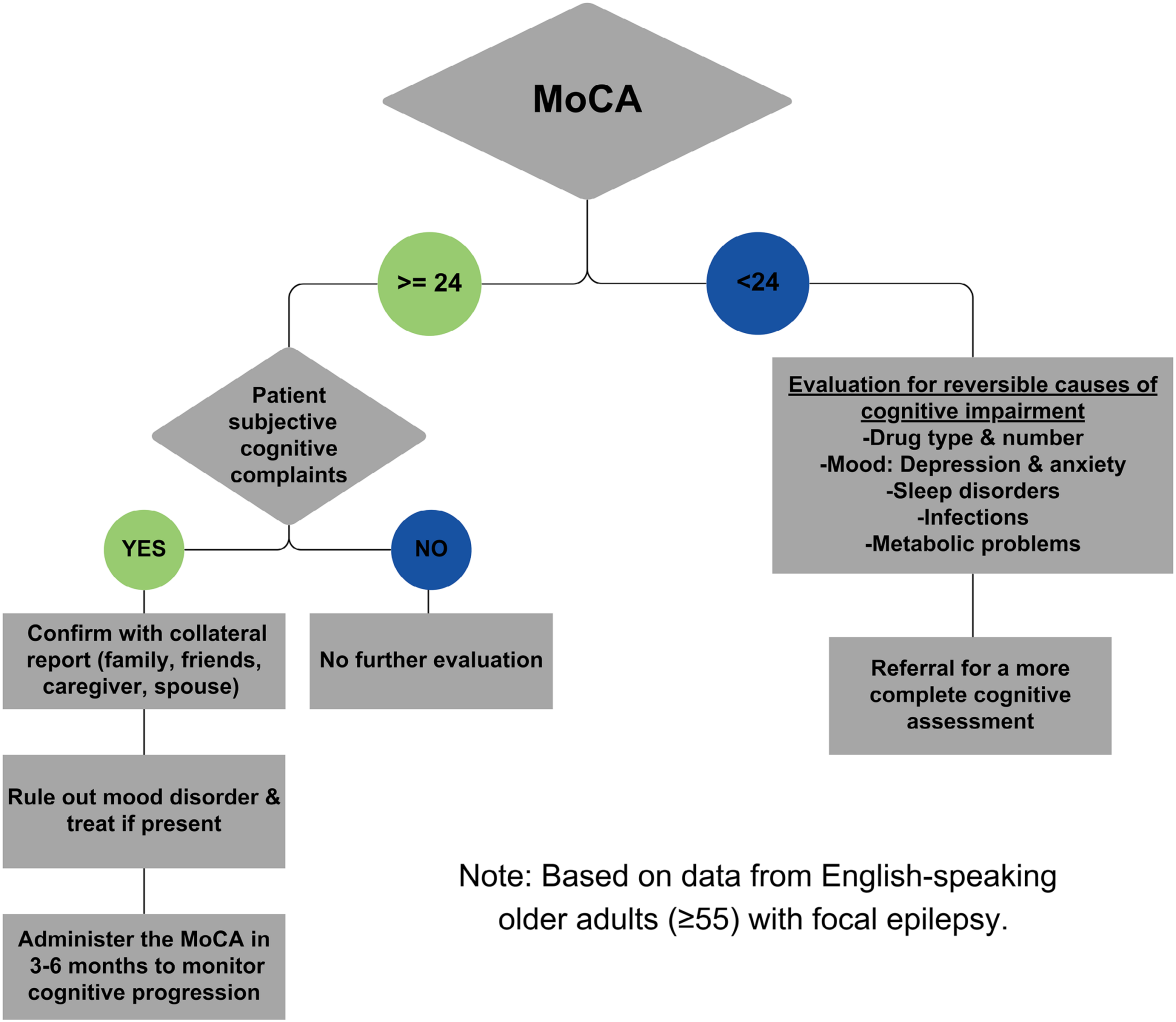
Decision tree for clinical evaluation using the MoCA. This decision tree may be used to determine if more comprehensive workup is needed based on the MoCA cutoff score. The cutoff proposed is based on sensitivity and specific analyses with older adults (age ≥ 55) with focal epilepsy.

Sensitivity, specificity, PPV, NPV, and Kappa statistics for a range of alternative cutoff scores on the MoCA are shown on Table 2. ROC plots are shown on Figure 2 with AUC values for each cutoff reported. All cutoff scores except for 27 and 28 had good AUC values (>0.70); however, only cutoffs 23 and 24 had a moderate agreement with the IC‐CoDE classification based on the Kappa statistic (Table 2). In our sample, 38% of patients had a MoCA total score < 24, and 22.6% had a score < 23. Of those patients with a MoCA <24, 63.2% were classified as IC‐CoDE *impaired*, including 26.4% with generalized impairment, 21.1% bi‐domain, 15.8% single domain, and the reminder 36.8% were classified as IC‐CoDE *intact*. Of those patients with a MoCA <23, 81.9% were classified as IC‐CoDE *impaired*, including 36.4% with generalized impairment, 36.4% bi‐domain, 9.1% single domain, and the reminder 18.2% were classified as IC‐CoDE *intact*. Figure 3 contains an evaluation decision tree based on a cutoff of <24.

### 3.5 Relationship between MoCA scores and demographic, clinical, and neuropsychological data

Table 3 shows Spearman rho correlations between MoCA total score and demographic and clinical variables for the entire sample. Greater years of education, lower number of ASM, and fewer errors on the ADAS‐Cog were all moderately associated with higher MoCA total score. Given that we did not find the previously reported relationship between age and MoCA total score, we conducted a post hoc partial correlation controlling for age of epilepsy onset (partial *r* = −0.205, *p* = 0.156). Table 3 shows Spearman rho correlations between individual neuropsychological tests and MoCA domain scores at the whole group level. Moderate associations were observed, with higher scores on letter fluency associated with higher scores on the abstraction domain; higher scores on animal fluency were associated with higher scores on the language domain; higher scores on auditory naming were associated with lower scores on the naming domain; and higher scores on memory domain were associated with higher scores on all three measures of memory (i.e., word list memory, story memory, and visual memory).

**TABLE 3:** (A) Correlations between MoCA total score and clinical, demographic, and global cognitive measure variables and (B) Correlations between MoCA domains and standard neuropsychological tests.

	Spearman rho	*p*
(A)		
Age	−0.156	0.278
Education	*0.340*	*0.016*
Age of epilepsy onset	0.028	0.848
Duration of epilepsy	−0.091	0.528
Number of ASM	*−0.283*	*0.049*
ADAS‐Cog	**−0.403**	**0.004**
(B)		
MoCA attention domain		
TMT‐A	0.192	0.181
Cancellation	0.121	0.401
MoCA abstraction domain		
TMT‐B	−0.096	0.508
Letter fluency	**0.414**	**0.003**
MoCA naming domain		
Visual naming (MINT)	0.251	0.082
Auditory naming (ANT)	**−0.334**	**0.018**
MoCA language domain		
Animal fluency	**0.316**	**0.025**
MoCA memory domain		
Word list memory (RAVLT‐LTPR)	**0.371**	**0.009**
Story memory (LMII)	**0.499**	**<0.001**
Visual delayed recall (VRII)	**0.620**	**<0.001**

*Note*: Bold: Significant with case‐wise FDR correction. Italics: Significant at <0.05.

Abbreviations: ADAS‐Cog, Alzheimer's Disease Assessment Scale–Cognitive Subscale; ANT, Auditory Naming Test; ASM, antiseizure medication; LMII, Logical Memory delayed recall; MINT, Multilingual Naming Test; RAVLT‐LTPR, Ray Auditory Verbal Learning Test Long‐term percent retention; TMT‐A, Trail Making Test Condition A; TMT‐B, Trail Making Test Condition B; VRII, Verbal Reproduction delayed recall.

Five factors emerged in the PCA with eigenvalues >1, accounting for 69.42% of the variance (Table S3). The first factor included story memory, word list memory, visual delayed recall, visual naming, and MoCA memory. The second factor included cancellation, auditory naming, and MoCA naming. The third factor included letter fluency, animal fluency, MoCA abstraction, and MoCA language. The fourth factor included word list memory, TMT‐B, and TMT‐A. The fifth factor included animal fluency and MoCA attention. For factor 2, auditory naming demonstrated a negative factor loading and for factor 5, animal fluency demonstrated a negative factor loading.

## 4 DISCUSSION

Cognitive abnormality has a known bidirectional relationship with epilepsy—present antecedent to epilepsy onset and diagnosis, evident at the time of diagnosis in drug naïve patients, with a longitudinal course of concern, particularly for those with severe drug‐resistant and/or symptomatic epilepsies^22^ as well as aging persons with new onset or lifelong chronic epilepsies.^3^ Critical treatment goals include identifying and characterizing cognitive abnormality, understanding the relative contribution of reversible versus non‐reversible causes, and working to minimize or prevent the adverse impact of cognitive morbidity on quality of life. In high‐resource countries, these efforts have been underway for some time but have been slowed in LMIC due to multiple barriers including but not limited to the fundamental issue of how best to efficiently screen for cognitive abnormality in the context of high demand and limited resources. As such, the rates and nature of cognitive disorders among patients with epilepsy residing in LMICs remain an understudied area, presenting a global public health issue as the majority of the world population of persons with epilepsy reside in these countries.^23^ In regions lacking validated comprehensive neuropsychological tests/norms or skilled neuropsychologists, clinicians reasonably rely on brief cognitive assessments to screen for cognitive impairment and dementia. In this investigation, we addressed the validity of the MoCA in older adults with epilepsy and addressed four key questions.

### 4.1 MoCA performance in overall sample

Overall, patients in our cohort had lower MoCA total scores compared to a large group of cognitively intact older adults. Patients who were classified as IC‐CoDE *impaired* had a lower MoCA total score and lower scores across the visuospatial/executive, naming, language, memory, and orientation domains. Patients with bi‐domain/generalized impairment demonstrated the lowest scores, followed by the single domain, and the intact group. In young‐to‐middle‐aged adults with epilepsy, lower MoCA scores have been reported compared to healthy controls.^24^, ^25^, ^26^, ^27^, ^28^ In older adults with epilepsy, Pirscoveanu et al.,^29^ reported lower scores on the MoCA relative to healthy controls, and other studies have reported a similar pattern using other cognitive screeners such as the MMSE^30^ and the Dementia Rating Scale.^31^ Furthermore, in this study, poorer performance on the MoCA was associated with more errors on the ADAS‐Cog, a commonly used and validated instrument to assess cognitive dysfunction in AD. Thus, our study provides additional evidence for the clinical utility and general sensitivity of this cognitive screener in older adults with epilepsy.

### 4.2 Overall accuracy of the MoCA compared to a contemporary cognitive taxonomic approach

Approximately one‐third of our cohort was classified as impaired based on the IC‐CoDE taxonomy, a similar rate to previous studies in older adults with epilepsy,^19^ compared to 54% classified as abnormal based on the MoCA (cutoff of <26). The recommended cutoff of <26 had an overall 72% accuracy, correctly classifying approximately 88% of the patients who were impaired based on the IC‐CoDE taxonomy. However, it failed to screen out 36.4% of the cognitively intact patients based on the actuarial criteria, suggesting false‐positive errors (i.e., inaccurate classification of cognitive impairment). Notably, patients who were incorrectly classified did not differ on important sociodemographic characteristics from those who were correctly classified. These findings align with previous validation studies reporting that the originally recommended cutoff score of 26 leads to a higher rate of false‐positives.^20^, ^21^, ^32^, ^33^ As such, a score less than <26 on the MoCA may help “screen” patients with epilepsy with significant cognitive impairment [dementia (i.e., major neurocognitive disorder)] and thus help initiate the diagnostic process that may include additional workup (e.g., lab tests, neuroimaging) and a more comprehensive neuropsychological assessment to identify the specific areas of impairment and to aid differential diagnosis (i.e., etiology of the cognitive deficits). However, for patients with more subtle deficits (i.e., mild cognitive impairment) or intact profiles, clinical judgment, and patient/family reports on recent cognitive or functional decline may be needed in addition to completing the MoCA (Figure 3). Furthermore, as recommended by Helmstaedter et al.,^34^ mood and adverse events should be screened and monitored over time, consistent with recommendations embedded in Figure 3. Relatedly, there has been a longstanding call to screen for mood disorders in epilepsy, particularly given the potential lethality of depression, and very brief screening tests that have been widely translated are available^35^ and can be reasonably extended to late onset epilepsies.

### 4.3 Optimal cutoff to detect cognitive dysfunction in older adults with epilepsy

Given that the original study published by Nasreddine et al.^16^ included a culturally homogenous, highly educated sample of healthy older controls and individuals with either MCI or mild AD,^16^ a large body of work has focused on validating alternative MoCA cutoffs across different medical populations, culturally and linguistically diverse samples, and individuals with lower levels of education. As such, we examined the sensitivity and specificity of several cutoff scores to derive an optimal cutoff score for our specific epilepsy population (i.e., older adults with epilepsy over age 55). After examination of several indices, a cutoff of 24 was optimal as it yielded an overall accuracy of 76% (compared to 72% using <26) and provided better specificity relative to the cutoff of <26. This suggests that the cutoff of 26 may overestimate the incidence of cognitive impairment. Although a cutoff score of 23 provided better accuracy (80%), sensitivity was lower relative to a cutoff of 24, suggesting higher false negative errors (i.e., classifying cognitively impaired patients as intact). Given that one of the primary purposes of a cognitive screener is to identify patients who may need further workup, higher sensitivity would be preferred to ensure that patients with potential cognitive impairment receive the proper clinical services. Alternative cutoff scores of 23 and 24 have been previously reported as having better diagnostic accuracy, and therefore, our findings corroborate these previous studies.^32^ Nonetheless, studies with larger samples are needed to compare the diagnostic performance of these two cutoff scores in older adults with epilepsy to drive an optimal cutoff that has clinical utility.

### 4.4 Associations between specific MoCA items and traditional neuropsychological measures

We were interested in examining whether MoCA domain scores relate to individual comprehensive neuropsychological tests. We found moderate to relatively strong associations between the MoCA memory domain and neuropsychological measures of list learning, story, and simple visual figure delayed recall, suggesting construct validity. Previous studies have also reported the strongest associations between neuropsychological measures of memory and the MoCA Memory domain.^36^, ^37^ Memory was the most impaired domain in our cohort based on the neuropsychological measures, providing some validation on the clinical utility of the MoCA as a memory screening tool. This is particularly relevant for older adults with epilepsy, as many of these patients demonstrate memory deficits and are at increased risk for dementia. Albeit modest, we also found correlations between category fluency and the MoCA language domain and letter fluency and the MoCA abstraction domain. An unexpected finding was an association between lower auditory naming scores and higher MoCA naming domain scores. Auditory naming had the lowest impairment rates in our cohort (6%), with the majority of the patients (76%) demonstrating average to above‐average performance. Thus, more variability in performance in our sample might have been needed to capture the variance in MoCA naming performance. These relationships were also observed using a PCA, where we found that MoCA domain scores demonstrated factor loadings with standard neuropsychological tests measuring similar cognitive constructs (i.e., memory tests and MoCA memory domain), providing further evidence of construct overlap between the MoCA and more comprehensive neuropsychological tests. These findings are comparable to Vogel et al.,^37^ where they found four factors using PCA, with MoCA domain scores loading to similar cognitive constructs as the standard neuropsychological tests. Similar to the correlational analyses, auditory naming demonstrated a negative factor loading, suggesting an inverse relationship with the MoCA naming domain. Although the attention domain did not correlate with other neuropsychological measures of attention; the attention domain loaded on the same factor as animal fluency, but an inverse relationship was observed. This may suggest that the naming and attention domains should be interpreted with caution as these scores are not showing expected associations with more comprehensive tests of the same cognitive construct. Notably, we did not have traditional measures of simple attention and working memory, such as digit span, which has been shown to be associated with performance on the attention and visuospatial/executive components.^37^ Nonetheless, there were no differences in performance between the IC‐CoDE *impaired* and *intact* groups on the attention, abstraction, and naming domains, suggesting that these components may be less reliable in identifying domain‐specific impairments in older adults with epilepsy. The individual MoCA domains had an overall classification accuracy of 78%, misclassifying approximately 23% of the impaired patients. The overall impairment classification rate for the MoCA domains (40%) was somewhat similar to the IC‐CoDE (34%), providing some evidence that the domains in the MoCA are sensitive to broader cognitive impairments beyond memory. The ability of the MoCA to detect non‐amnestic cognitive changes increases its clinical utility in epilepsy, as the cognitive profiles observed are broader compared to many other neurological conditions.^14^, ^22^, ^38^, ^39^, ^40^

### 4.5 Impact of important sociodemographic and clinical factors on MoCA performance

Individuals with epilepsy have fewer years of education compared to the general population, which has been associated with greater cognitive deficits. Better performance on the MoCA has been associated with greater years of education,^41^ and therefore, an education adjustment for individuals with lower levels of education (i.e., less than 12 years) has been recommended. Although only 6% of our cohort required the education adjustment, greater years of education continued to be correlated with higher MoCA total scores. Notably, there were no differences in education between the IC‐CoDE i*mpaired* and *intact* groups; however, many of our measures were adjusted for education (Table S2). There are several normative datasets available for the MoCA that adjust for the effects of age, education, or sex on performance,^41^, ^42^, ^43^, ^44^ which can be used in combination with the MoCA cutoffs. Despite our cohort representing a well‐seizure‐controlled sample taking on average 1–2 ASM, although modest, there was an association between a greater number of ASM and lower scores on the MoCA. A greater number of ASM has been associated with a worse cognitive profile using standard neuropsychological testing, and in older adults with idiopathic epilepsy has been associated with lower scores on the MoCA.^29^ This provides further evidence of the clinical utility of the MoCA as a cognitive screening tool for epilepsy. Interestingly, we did not find an association between age and total MoCA Score as has been widely reported in other studies,^41^, ^45^ with younger individuals demonstrating higher scores. Notably, the relationships between cognitive performance and demographic variables (e.g., age, education) that are commonly observed in healthy controls are inconsistently found in adults and children with epilepsy, likely due to disease and treatment‐related factors. Given that our cohort comprised patients with early and late‐onset epilepsy, we examined the relationship between age and MoCA total scores controlling for age of epilepsy onset. However, the association did not reach significance. It is possible that the previously reported relationship between age and MoCA performance may be observed in those with late‐onset epilepsy but disrupted in patients with early onset; however, larger samples are needed.

### 4.6 Limitations of the MoCA and its use

While offering several advantages as a cognitive screening measure for older adults with epilepsy, its limitations should be recognized. First, the MoCA is not a substitute for traditional neuropsychological assessment in that it does not assess a number of important cognitive skills, and for those abilities it does assess, it contains a limited number of items per “domain” which can impact test reliability. Second, the MoCA was devised as a screening measure for older adults, yet it has been used in the epilepsy literature to assess cognition in younger persons with epilepsy, a use for which the reliability and validity of the test remains to be determined. Third, clinic volumes can be extraordinarily high, with limited patient contact time in many parts of the world, presenting surprising challenges for the use of even brief cognitive screening measures. The application of machine learning procedures to predict outcomes of cognitive screening tests such as the MoCA using sociodemographic and clinical epilepsy factors suggests an added layer of research that could advance cognitive care in high volume/low resource settings.^46^ Fourth, our results were derived from a sample of older epilepsy patients from a high‐resource country with a modest number of participants, thus the generalizability of the findings to other samples remains to be determined. However, we believe the analytic steps taken are generalizable and, in fact, fundamental for the task of determining the clinical utility of the MoCA as a screening tool for epilepsy across other countries. Lastly, studies with a wider age and education range are needed to explore optimal cutoffs stratified by age and education.

Nonetheless, our study has several strengths including the validation of cognitive impairment based on a well‐established cognitive taxonomy for epilepsy, administration of the MoCA and the standard neuropsychological measures during the same testing session, and a relatively homogenous epilepsy cohort. We add to this literature by demonstrating that the MoCA is a valuable screening tool that can be used to guide clinical decision‐making in epilepsy and help determine if a comprehensive neuropsychological evaluation is warranted.

## CONFLICT OF INTEREST STATEMENT

None of the authors has any conflict of interest to disclose.

## ETHICS STATEMENT

We confirm that we have read the Journal's position on issues involved in ethical publication and affirm that this report is consistent with those guidelines.

## Supporting information


Tables S1–S3

## Data Availability

Authors have full access to all study data and participant consent forms and take full responsibility for the data, the conduct of the research, the analyses and interpretation of the data, and the right to publish all data. The data supporting the findings of this study are available on request from the senior author (C.R.M.).
