# Supplementary material for: Validity of the MoCA as a cognitive screening tool in epilepsy: Are there implications for global care and research?
Source: Epilepsia Open. 2024 Jun 14;9(4):1526–37. doi: 10.1002/epi4.12991 (PMC11296095; doi:10.1002/epi4.12991)
Supplement: Supplementary file 1 — Tables S1–S3 [file EPI4-9-1526-s001.docx]

| **Supplemental Table 1: Epilepsy Investigations Using the MoCA** | | |
| --- | --- | --- |
| **Investigators** | **Country** | **Focus** |
| Sharma et al. (2024 | USA | Association between cognitive performance and gray matter volumes. |
| Mamo et al. (2024) | Ethiopia | Cognitive screening in epilepsy and predictors of medication adherence. |
| Aboseif et al. (2024) | USA | Longitudinal disability in autoimmune epilepsy. |
| Huang et al. (2023) | China | Topographical distribution of sleep spindles with varying degree of cognitive impairment. |
| Ochoa-Morales et al. (2023) | Mexico | Predictors of internalized stigma. |
| Liu et al. (2023) | China | Cognitive outcome of generalized convulsive status epilepticus treated with phenobarbital vs valproate. |
| Huang et al. 2023) | China | Relationship between cognitive function with sleep spindle characteristics. |
| Ueda et al. (2023) | Japan | Neurofilament light chain and cognition. |
| Yang et al. (2023) | China | Clinical trial of transcutaneous auricular VNS for treatment of drug resistant epilepsy (DRE). |
| Novak et al. (2023) | Slovenia | Cognitive screening in epilepsy. |
| Almwled et al. (2022) | Saudi Arabia | Predictors for epilepsy medication adherence. |
| Hou et al. (2022) | China | Cognitive impairment risk factors in post-stroke epilepsy. |
| Rezakhani et al. (2022) | Iran | Efficacy of transcranial direct-current stimulation in DRE. |
| Chang et al. (2022a) | China | Degree centrality and functional connectivity in temporal lobe epilepsy with ictal panic |
| Chang et al (2022b) | China | Resting state fMRI in temporal lobe epilepsy with ictal panic. |
| He et al. (2021) | China | Presence and predictors of cognitive impairment in TLE |
| De Souza et al (2021) | Brazil | Comparison of screening tests to assess cognition. |
| Popp et al. (2021) | USA | Predictors of cognitive impairment in IGE and relationship with exercise. |
| Lin et al. (2021) | China | Use of machine learning to identify predictors of cognitive abnormality. |
| Fonseca et al. (2021) | Spain | Sleep quality and cognitive status. |
| Zhong et al. (2021a) | China | Predictors of depression in new onset epilepsy over 12 months. |
| Zhong et al. (2022b) | China | Predictors of depression in established epilepsy. |
| Zhao et al. (2021) | China | Morphometric and cognitive change after anterior temporal lobectomy. |
| Wu et al. (2021) | China | Binocular rivalry in idiopathic generalized epilepsy and association with clinical factors and cognition. |
| Montano-Loxada et al. (2021) | Colombia | Examine cognition and predictors or cognition in epilepsy outpatients. |
| Njamnshi et al. (2020) | Cameroon | Predictors of cognition in onchocerciasis-endemic rural community. |
| Masoudian et al. (2020) | Iran | Examine cognition in generalized epilepsy compared to non-epileptic neurological diseases. |
| Oustad et al. (2020) | Iran | Effects of donepezil and memantine on cognitive disorder In TLE. |
| Karaaslan & Hamamci (2020) | Turkey | Cognitive status of epilepsy and PNES compared to controls. |
| Rodrigues et al. (2020) | Portugal | Monitoring of a patient with absence status epilepticus. |
| Lin et al. (2020) | China | Cognition in focal epilepsy with focal cortical dysplasias –graph theoretical analysis. |
| Liu et al. (2020) | China | Impact of sodium valproate plus levetiracetam in children with epilepsy |
| Harby et al. (2020) | Egypt | Levetiracetam concentration in peripheral blood mononuclear cells and clinical outcomes. |
| Wang et al. (2020) | China | Predictors of cognitive impairment. |
| Zang et al. (2020) | China | Predictors of postictal cognitive function |
| Feter et al. (2020) | Brazil | Impact of exercise program on cognition. |
| Gavrilovic et al. (2019) | Serbia | Effects of clinical seizure features on cognitive course. |
| Li et al. (2019) | China | NR3B antibodies and cognition. |
| Zacharia & Eslinger (2019) | US | fMRI cerebellar activation patterns in epilepsy with brain tumor. |
| Del Brutto et al. (2019) | Ecuador | Predictors of cognition in patients with neurocysticercosis. |
| Xu et al. (2018) | China | White matter imaging correlates of cognition. |
| Zhang et al. (2018) | China | Alterations in resting state networks following ATL and cognition. |
| Yang et al. (2018) | China | Resting state patterns in TLE and cognition. |
| Yang et al. (2018) | China | Predictors of cognition in youth with epilepsy. |
| Zhou et al. (2017) | China | Efficacy of gastrodin folate and vitamin B12 in post-stroke epilepsy cognition. |
| Zang et al. (2017) | China | Rs-fMRI intractable unilateral TLE with impaired executive control function |
| Rahman et al. (2015) | Australia | Cognitive function in patients with brain tumor related epilepsy. |
| Hanby et al. (2015) | UK | Structural and physiological MRI correlates of cerebrovascular disease in late onset epilepsy. |
| Jiang et al. (2014) | China | Empathy and emotion recognition effects on cognition. |
| Phabphal & Kanjanasartien (2011) | Thailand | Presence and predictors of cognitive impairment. |
| Pirscoveanu et al (2010) | Romania | Cognition in elderly with epilepsy |

| Supplementary Table 2: Neuropsychological Tests and normative data | | | | |
| --- | --- | --- | --- | --- |
| Test | Description | IC-CoDE Domain | Demographic Corrections | Normative Data |
| Rey Auditory Verbal Learning Test (RAVLT) Long Term Percent Retention | RAVLT consist of five learning trials of 12 words and a recall trial of the word list after a 30-minute delay. A long-term retention score is calculated by dividing delayed recall by Trial 5 score (RAVLT- LTPR). | Memory | Age | Mayo Older Americans Normative Study^1^ |
| Wechsler Memory Scale 4^th^ Edition (WSM-4) Logical Memory (LM) Story B delayed recall | The examinee is read a short story and asked to recall it immediately and after a 20-30-minute delay (LMII). Total score is the number of story elements recalled. | Memory | Age | Mayo Older Americans Normative Study^1^ |
| WMS-4 Visual Reproduction (VR) delayed recall | The examinee is shown a series of designs and after each stimulus presentation they are instructed to draw the designs from memory. After a 20–30-minute delay, the examinee is instructed to draw as many of the designs from memory as possible (VRII). | Memory | Age | Test mannual^2^ |
| Multilingual Naming Test (MINT) | The MINT consists of a series of 32-line drawings and examinees are instructed to name the object in each drawing; the MINT score is the total number of drawings correctly identified. | Language | Age, education, and sex | Uniform Data Set (UDS) noms^3^ |
| Auditory Naming Test (ANT) | The ANT consist of a series of 36 auditory descriptions of items and examinees are instructor to provide the name of the item being described; the ANT score is the total number of item descriptions correctly identified. | Language | Age and education | Hamberger et al. (2022)^4^ |
| Semantic Fluency | Examinees are instructed to generate as many animals as possible in 60 seconds; total score is the total number of animals provided. | Language | Age, education, sex, and race | Expanded Halstead-Reitan Battery^5^ |
| Trail-Making Test Condition A (TMT-A) | Examinees are instructed to connect numbers in ascending order as quickly as possible; score is completion time in seconds. | Processing Speed/  Attention | Age, education, sex, and race | Expanded Halstead-Reitan Battery^5^ |
| Alzheimer’s Disease Assessment Scale–Cognitive Subscale (ADAS-Cog) Number Cancellation subtest | Examinees are instructed to cross off as many target digits as possible in 45 seconds; total score is number of target hits minus number of errors. | Processing Speed/  Attention | Based on a demographically-matched control group | ADNI Normal Control Sample^6^ |
| Letter Fluency | Examinees are given 60 seconds to generate as many words as possible beginning with the letters F, A, and S; score is the total number of words provided across all three letters. | Executive Function | Age, education, sex, and race | Expanded Halstead-Reitan Battery^5^ |
| Trail-Making Test Condition B (TMT-B) | Participants are instructed to alternate between connecting numbers and letters in ascending order as quickly as possible; score is completion time in seconds | Executive Function | Age, education, sex, and race | Expanded Halstead-Reitan Battery^5^ |
| ^1^ Ivnik RJ, Malec JF, Smith GE, Tangalos EG, Petersen RC, Kokmen E, Kurland LT. Mayo's older Americans normative studies: updated AVLT norms for ages 56 to 97. The Clinical Neuropsychologist. 1992;6(S1):83-104.  ^2^ Wechsler D. WMS-IV: Wechsler memory scale: PsychCorp; 2009.  ^3^ Weintraub S, Besser L, Dodge HH, Teylan M, Ferris S, Goldstein FC, et al. Version 3 of the Alzheimer Disease Centers’ neuropsychological test battery in the Uniform Data Set (UDS). Alzheimer disease and associated disorders. 2018;32(1):10.  ^4^ Hamberger MJ, Heydari N, Caccappolo E, Seidel WT. Naming in older adults: Complementary auditory and visual assessment. Journal of the International Neuropsychological Society. 2022;28(6):574-87.  ^5^ Heaton R, Miller SW, Taylor MJ, Grant-Isibor I. Revised comprehensive norms for an expanded Halstead-Reitan Battery: Demographically adjusted neuropsychological norms for African American and Caucasian adults. 2004.  ^6^Cancellation raw scores were converted into z-scores based on data from a sample of 370 cognitively normal older adults from the Alzheimer's Disease Neuroimaging Initiative (ADNI) database (adni.loni.usc.edu). | | | | |

| Supplementary Table 3: Factor loadings based on principal component analysis | | | | | |
| --- | --- | --- | --- | --- | --- |
|  | Component | | | | |
| Variable | 1 | 2 | 3 | 4 | 5 |
| Story Memory (LMII) | 0.779 |  |  |  |  |
| Word List Memory (RAVLT-LTPR) | 0.398 |  |  | 0.64 |  |
| Visual Delayed Recall (VRII) | 0.833 |  |  |  |  |
| TMT-B |  |  |  | 0.89 |  |
| Letter Fluency |  |  | 0.683 |  |  |
| TMT-A |  |  |  | 0.66 |  |
| Cancellation |  | 0.546 |  |  |  |
| Animal Fluency |  |  | 0.434 |  | -0.65 |
| Visual Naming (MINT) | 0.565 |  |  |  |  |
| Auditory Naming (ANT) |  | -0.734 |  |  |  |
| MoCA Attention |  |  |  |  | 0.80 |
| MoCA Abstraction |  |  | 0.776 |  |  |
| MoCA Naming |  | 0.809 |  |  |  |
| MoCA Language |  |  | 0.768 |  |  |
| MoCA Memory | 0.788 |  |  |  |  |
| Eigenvalue | 4.620 | 1.732 | 1.552 | 1.369 | 1.141 |
| Proportion of variance (rotated), % | 18.74 | 14.48 | 14.27 | 13.58 | 8.35 |
| Extraction Method: Principal Component Analysis.  Rotation Method: Varimax with Kaiser Normalization.  Factor Loadings <0.350 were suppressed from the Table. | | | | | |
